# The impact of ADHD and conduct disorder in childhood on adult delinquency: A 30 years follow-up study using official crime records

**DOI:** 10.1186/1471-244X-11-57

**Published:** 2011-04-11

**Authors:** Marianne Mordre, Berit Groholt, Ellen Kjelsberg, Berit Sandstad, Anne Margrethe Myhre

**Affiliations:** 1 Division of Mental Health and Addiction, Oslo University Hospital, Norway; 2 Institute for Clinical Medicine, University of Oslo, Norway; 3 Centre for Forensic Psychiatry, Division of Mental Health and Addiction, Oslo University Hospital, Norway; 4 Unit of Biostatistics and Epidemiology, Oslo University Hospital, Norway

## Abstract

**Background:**

Few longitudinal studies have explored lifetime criminality in adults with a childhood history of severe mental disorders. In the present study, we wanted to explore the association between adult delinquency and several different childhood diagnoses in an in-patient population. Of special interest was the impact of disturbance of activity and attention (ADHD) and mixed disorder of conduct and emotions on later delinquency, as these disorders have been variously associated with delinquent development.

**Methods:**

Former Norwegian child psychiatric in-patients (n = 541) were followed up 19-41 years after hospitalization by record linkage to the National Register of Criminality. On the basis of the hospital records, the patients were re-diagnosed according to ICD-10. The association between diagnoses and other baseline factors and later delinquency were investigated using univariate and multivariate Cox regression analyses.

**Results:**

At follow-up, 24% of the participants had been convicted of criminal activity.

In the multivariate Cox regression analysis, conduct disorder (RR = 2.0, 95%CI = 1.2-3.4) and hyperkinetic conduct disorder (RR = 2.7, 95% CI = 1.6-4.4) significantly increased the risk of future criminal behaviour. Pervasive developmental disorder (RR = 0.4, 95%CI = 0.2-0.9) and mental retardation (RR = 0.4, 95%CI = 0.3-0.8) reduced the risk for a criminal act. Male gender (RR = 3.6, 95%CI = 2.1-6.1) and chronic family difficulties (RR = 1.3, 95% CI = 1.1-1.5) both predicted future criminality.

**Conclusions:**

Conduct disorder in childhood was highly associated with later delinquency both alone or in combination with hyperactivity, but less associated when combined with an emotional disorder. ADHD in childhood was no more associated with later delinquency than the rest of the disorders in the study population. Our finding strengthens the assumption that there is no direct association between ADHD and criminality.

## Background

Knowledge about which child psychiatric disorders precede criminal behaviour is important to delineate high risk children seen in child psychiatric services. Research has consistently demonstrated the long-term impact of childhood psychiatric problems on later antisocial traits, especially conduct problems that have been shown to be developmental precursors of later antisocial behaviour and criminality [1-8]. Recent studies conducted on prisoners in western countries have shown that about half of the imprisoned fulfilled the diagnoses of serious conduct disorder or antisocial personality disorder when incarcerated [9,10].

Although conduct disorder is a well known antecedent of antisocial development, other childhood disorders as precursors of antisociality are more controversial. So far, long-term follow-up studies have demonstrated that attention-deficit/hyperactivity disorder (ADHD) combined with conduct disorder is a precursor of later antisocial behaviour [11-13]. There are, however, discrepant findings with regard to ADHD without conduct problems as an independent precursor of criminality. Based on the results of long-term epidemiological follow-up studies, Farrington [3], Babinski et al. [14] and Sourander et al. [8] found that hyperactivity-impulsivity, independently of conduct problems, predicted later criminality in males. In two long-term clinical follow-up studies, Mannuzza et al. [15,16] similarly found that ADHD was a developmental precursor of antisocial behaviour in early- and mid-adulthood. Satterfield et al. [13], on the other hand, reported in his clinical follow-up study of hyperactive outpatient boys that only those individuals with ADHD combined with childhood conduct problems were at increased risk of criminality. Likewise, in a 10-year follow-up study of a birth cohort, Fergusson et al. found that children with attention deficits but no conduct problems were not at increased risk of juvenile delinquency. They were, however, at risk of later reduced academic success in a dose-response manner [17]. Recently, Diamantopolou et al. [2], similarly, found that there were no direct association between ADHD symptoms and later antisocial personality problems. Neither did they find that the combination of internalizing and externalizing symptoms appeared to add to the prediction of later antisocial behaviour in adolescence.

During the last decades, there has been a growing interest in the interplay between internalizing and externalizing problems, but there have been no clear findings about the outcome for children with comorbid conduct and emotional disorders. Sourander et al. found that children with combined emotional and conduct problems had a higher risk of criminality compared with children who only had emotional problems, attention deficits and/or conduct problems [8]. Their results provided only partial support from previous research. In two longitudinal clinical studies, Harrington et al. [18] and Fombonne et al. [19] found that children and adolescents with comorbid conduct and depressive disorders had a higher risk of later criminality and antisocial behaviour than those who only had emotional disorders. However, the outcome among children with comorbid disorders was similar to those with conduct disorders alone.

In sum, there are still no consistent findings from epidemiological or clinical studies whether ADHD alone is a precursor of later criminality; nor is it known whether children with combined emotional and conduct disorder are at higher risk of later antisocial behaviour than those with conduct disorder alone or in combination with hyperactivity.

Studying child psychiatric in-patients with excessive symptom load could enhance prediction of which disorders precede criminality. Previous research has shown that severity of symptoms increases the stability of a disorder [20], and clinical referred children have been found to have high diagnostic stability from childhood to adolescence [21]. To our knowledge, there are, however, few long-term follow-up studies of seriously affected in-patient children with ADHD or comorbid emotional and conduct disorder. Only two of the previously mentioned clinical studies had included in-patients [18,19].

In the present study, former child psychiatric in-patients were followed up 19 to 41 years after hospitalization by linking their records to the National Register of Criminality. The combination of a long follow-up period and diagnostic evaluation, according to the ICD-10 classification system, made this study suited for exploring the association between several different childhood diagnoses and the development of criminality in adolescence and into mid-adulthood. The mean age at follow-up was 38 years, an age after which the likelihood of criminal debut is minimal. We could, therefore, provide a comprehensive picture of lifetime criminality in adults with a childhood history of severe mental disorders.

In addition, the extensive information in the hospital records made it possible to control for vulnerability factors, other than diagnoses, that could contribute to the development of later criminality.

We wanted to test the hypothesis that there was a direct association between hyperkinetic symptoms and later criminality in former child psychiatric in-patients, with ADHD increasing the risk for delinquency, independent of conduct disorder comorbidity or not.

We also wanted to test the hypothesis that former child psychiatric in-patients with mixed disorder of conduct and emotions were at increased risk for later criminality compared to those with conduct disorder only. A final issue was to explore whether vulnerability factors other than diagnoses could enhance prediction of delinquent outcome.

## Methods

### Procedure

All consecutively admitted in-patients to the children's unit at the National Centre for Child and Adolescent Psychiatry (NCCAP), in Oslo Norway, from January 1968 to October 1988, were included in the study (n = 635). With regard to ethnicity, all but ten were Caucasians. The NCCAP's children's unit, which was opened in 1968, provided specialized treatment for children 13 years or younger from all over Norway who had complex child psychiatric disorders in need of skilled treatment. The hospital records provided detailed baseline information of behaviour and symptoms, psychological test results, school performance (all children of school age had adjusted school programmes during their hospitalization) and extensive anamnestic information about the children and their families. The study population was identified from the population register at the Central Bureau of Statistics, by using the citizen's identity number, which ensured a definite identification. The patient group was matched with the National Register of Criminality at follow-up in July 2007. The criminal register has lifetime information about all criminal proceedings against everyone residing in Norway. The reported findings are based on court convictions for infractions of all breaches of the law. The terms "delinquent", "criminal" and "convicted" are used interchangeably in this paper.

Age at first and last entry into the register was recorded, together with a description of the offences committed. The offences were classified into violent offences (all offences involving interpersonal aggression and threats, robbery, arson), sexual offences (offences against public decency, immoral intercourse with minors, incest, rape), crimes against property (larceny of all kind, frauds, forgery, embezzlement), drug violations and "other offences" (traffic offences, vandalism, possession of weapons, refusal to obey orders, vagabonding, crimes against military law). The sentences were categorized in terms of judicial fine only, conditional imprisonment, unconditional imprisonment and mandatory care. Under the Norwegian criminal code, people with mental retardation are able to stand trial. People with mild mental retardation (IQ between 70-55) can be sentenced to ordinary prisons. Offenders in the category of severe mental retardation (IQ below 55) are seldom prosecuted, but can be sentenced to mandatory care for a period of 3 years [22].

### Participants

For 635 in-patients admitted to hospital, 78 of the hospital records could not be located. In one case, the record was incomplete, and another patient who was older than 13 years at admission was excluded. In five cases, we could not determine the personal identification number at the Central Bureau of Statistics.

A total of 550 subjects (87% of the original sample) were identified in the population register at the Central Bureau of Statistics at follow-up in 2007. Of these, 25 (5%) had died and 14 (3%) had emigrated. Those who had emigrated or died before the age of 14 years (n = 9), which was the legal age of criminal responsibility at that time, were excluded from the study. Thus, a total of 541 participants were included in this study.

The sex distribution was 366 (68%) boys and 175 (32%) girls, and the mean age at hospitalization was 7.9 years (SD 2.7, range 0-13).

The mean age at follow-up (when those who had emigrated or died were excluded) was 38.3 years (SD 7.0, range 23-52), and the mean follow-up period from first admission was 30.4 years (SD 6.6, range 19-41).

With regard to treatment, 57% of the patients were admitted to the family ward, where the intervention was based mainly on diagnostic evaluation and family therapy. The other patients, who were admitted to the inpatient long-term ward (40%) and to the day care ward (3%), received diagnostic evaluation, psychodynamic-oriented individual therapy and/or social psychiatric interventions. The mean length of stay was 1.1 months at the family ward, 8.2 months at the inpatient long-term ward and 22.5 months at the day care ward. In total, 24% of the in-patients were admitted more than once.

### Measures

#### Mental health (ICD-10)

Based on all the information in the hospital records, including weekly ward descriptions of the children, all the patients were re-diagnosed according to current criteria in ICD-10 [23]. The hospital records were comprehensive with extensive anamnestic information provided by parents, teachers and local health workers. All 541 patients were re-diagnosed by the first author and independently by at least one other experienced child psychiatrist. If the two raters disagreed, the case was discussed by a research group of four child psychiatrists, and a consensus diagnosis was established.

It was found that 25% of the patients had more than one psychiatric ICD-10 diagnosis, with nonorganic eneuresis or encopresis being the co-diagnoses most often encountered (59% of the cases). The diagnosis of greatest clinical importance (principal diagnosis) was pre-empted in this study. Table 1 contains a summary of the principal diagnoses, which were clustered into 10 groups: 1) **Conduct disorder **(F91); 2) **Disturbance of activity and attention/ADHD **(F90.0), (in accordance with ICD-10 diagnostic criteria, they all fulfilled the DSM-IV criteria for the corresponding ADHD of combined type, except for five participants who fulfilled the criteria for ADD); 3) **Hyperkinetic conduct disorder (**(F90.1), the criteria for both hyperkinetic and conduct disorders must be met to achieve the diagnosis); 4) **Mixed disorder of conduct and emotions (**(F92), the criteria for both an emotional disorder and a conduct disorder must be met to achieve the diagnosis); 5) **Emotional disorder**, including emotional disorders in childhood (F93), anxiety and other neurotic disorders (F40-F49), mood disorders (F30-F39), eating disorders (F50) and mutism (F94.0); 6) **Attachment disorder **(F94.1 and F94.2); 7) **Pervasive developmental disorder **(PDD) (F84); 8) **Mental retardation only (MRO) **(F70-F79 as the only diagnosis); 9) **Other disorders**, including organic mental disorders (F06), tic disorders (F95), nonorganic eneuresis (F98.0), encopresis (F98.1), stuttering (F98.5) and psychosis (F20); and 10) **Z-group diagnoses **including diagnoses given for factors influencing health status and contact with health services. Investigations of problems within the family usually led to such a diagnosis. Descriptions of the child's symptoms did not meet the criteria for a psychiatric diagnosis.

**Table 1:** Distribution and descriptive characteristics of diagnostic groups at admission

Diagnostic groups	N	Male gender N (%)	CFD Mean (SD)	CGAS Mean (SD)	N^C^*	MR N (%)
Conduct disorder (F91)	45	37 (82)	4.7 (1.0)	43.5 (6.4)	43	1 (2)

Disturbance of activity and attention/ADHD (F90.0)	40	30 (75)	3.4 (1.6)	41.4 (7.1)	36	13 (36)

Hyperkinetic conduct disorder (F90.1)	46	39 (85)	4.1 (1.4)	40.0 (4.1)	43	7 (16)

Mixed disorder of conduct and emotions (F92)	78	55 (71)	4.6 (1.1)	42.8 (6.5)	76	9 (12)

Emotional disorder (F30, F40, F50, F93, F94.0)	121	60 (50)	4.1 (1.4)	47.2 (10.1)	117	15 (13)

Attachment disorder (F94.1, F94.2)	20	12 (60)	5.4 (0.9)	39.6 (2.8)	18	3 (17)

PDD (F84)	110	88 (80)	3.2 (1.4)	31.7 (6.2)	107	71 (66)

Mental retardation **only **(F70)	29	20 (69)	3.1 (1.6)	34.0 (5.9)	29	29 (100)

Residual disorders (F06, F20, F95, F98.0, F98.1, F98.5)	33	14 (42)	3.7 (1.4)	36.0 (13.0)	32	20 (63)

Z-group diagnoses	19	11 (58)	4.7 (0.9)	69.5 (13.5)	16	2 (13)

Total study population	541	366 (68)	4.0 (1.4)	41.2 (11.1)	517	170 (33)

N^C^* = 24 records were too incomplete to assess cognitive abilities, and were recorded as missing.

#### Socio-demographic variables

Gender was registered at baseline and reported in table 1.

We also applied a global assessment of chronic family difficulties (CFD) [24] based on all the information available in the hospital records of the past and present family situation. Socioeconomic conditions, social network, marital or family discord and current/previous physical and mental health of the family members were recorded. The total burden of difficulties was scored on an interval scale from 0 to 6. A score of 0 reflects no sign of chronic family difficulties and a score of 6 reflects severe difficulties/very disturbed family environment (Table 1).

#### Level of cognitive abilities

An assessment of each participant's cognitive level was based on all the information available in the hospital records, including clinical findings, psychometric test results (in some cases standardized intelligence tests, e.g. Wechsler Intelligence Scale for Children (WISC), Standford-Binet Intelligence Scales, Leiter International Performance Scale) and pedagogic tests (e.g. Illinois Test of Psycholinguistic Abilities (ITPA), Peabody Picture Vocabulary Test) during hospitalization. For children of school age, systematic pedagogical evaluations were performed by teachers at NCCAP's affiliated school. Diagnostic criteria for mental retardation were used according to the ICD-10. In the present study, cognitive level was dichotomized in terms of mental retardation (MR) yes/no, which correspond to the approximate cut-off for IQ greater or less than 70 (Table 1). Previous research has shown that having an IQ of at least 70 is an important prognostic factor for delinquency often used in the literature [22,25,26]. In 24 cases, lack of information in the records made it impossible to assess cognitive functioning, and so the corresponding data were recorded as missing.

#### Children's Global Assessment Scale (CGAS)

The children were also reassessed on the CGAS, a global assessment of the child's psychosocial functioning [27]. The scale runs from 1 to 100, with 1 indicating the most severely disordered and 100 the best functioning child. The assessment was based on the child's functioning at admission (Table 1).

### Inter-rater reliability study

An inter-rater reliability study was carried out for 476 patients, yielding an overall kappa value of 0.77 for the ICD-10 diagnoses in Table 1 (varying from 0.52 for attachment disorders to 0.89 for PDD and mental retardation), and intraclass correlation coefficients (ICC) of 0.83 for CGAS, 0.86 for CFD and 0.85 for cognitive level.

### Statistical methods

Descriptive statistics are presented as means with standard deviations, medians and ranges, as appropriate. Variables were investigated using Student's two-sample *t *test for continuous variables, and Pearson's chi-square test and Fisher's exact test for categorical variables. Cox proportional regression analyses were used to analyse the risk of later convictions. In these analyses, participants were followed from the age of 14 years, which was the youngest age for registration of criminality, until their first contact with the police, or otherwise, their date of emigration, death, or else their follow-up in July 2007 for those who had not been convicted. The effects of possible prognostic variables were tested using univariate Cox regression. Variables that were significant at the 5% level were included in a multivariate Cox regression analysis. Hazard ratios, which were used as measures of relative risk, are presented together with their 95% confidence intervals.

Kappa statistics and intraclass correlation coefficients (ICC) analyses were used to examine the inter-rater reliability.

SPSS version 15 was used for the statistical analyses.

### Ethics

The study was approved by the Regional Committee of Ethics in Medical Research, the Department of Health and Social Services and the Norwegian Data Inspectorate.

## Results

### Crime rates

Of the total sample of 541 persons, 131 (24%) were found in the crime registry at follow-up (Table 2). Of these, 114 (31%) of the males and 17 (10%) of the females had been convicted. Of the 131 individuals who committed crimes, 85 (65%) were re-offenders.

**Table 2:** Vulnerability factors for delinquency.

Vulnerability factors	N = 541	Non-convicted N = 410	Convicted N = 131	Unadjusted	Adjusted
		**N (%)/Mean(SD)**	**N (%)/Mean (SD)**	**RR (95% CI)**	**RR (95% CI)**

**Mental health (ICD-10)**					

Conduct disorder (F91)	45	20 (44)	25 (56)	3.2 (2.0-4.9)***	2.0 (1.2-3.4)*

Disturbance of activity and attention/ADHD (F90.0)	40	29 (73)	11 (27)	1.2 (0.7-2.2)	-″

Hyperkinetic conduct disorder (F90.1)	46	21 (46)	25 (54)	3.4 (2.2-5.2)***	2.7 (1.6-4.4)***

Mixed disorder of conduct and emotions (F92)	78	52 (67)	26 (33)	1.6 (1.1-2.5)*	ns

Emotional disorder (F30, F40, F50, F93, F94.0)	121	96 (79)	25 (21)	0.8 (0.5-1.2)	-″

Attachment disorder (F94.1, F94.2)	20	12 (60)	8 (40)	1.9 (0.9-3.9)	-″

PDD (F84)	110	103 (94)	7 (6)	0.2 (0.1-0.4)***	0.4 (0.2-0.9)*

Mental retardation **only **(F70)	29	28 (97)	1 (3)	0.1 (0.0-0.9)*	▫

Residual disorders (F06, F20, F95, F98.0, F98.1, F98.5)	33	30 (91)	3 (9)	0.3 (0.1-1.0)	-″

Z - group diagnoses	19	19 (100)	0	0.1 (0.0-3.4)	-″

**Sociodemographic variables**					

Male gender	366	252 (69)	114 (31)	3. 8 (2.3-6.3)***	3.6 (2.1-6.1)***

Chronic family difficulties scale		3.8 (SD 1.4)	4.5 (SD 1.4)	1.4 (1.2-1.6)***	1.3 (1.1-1.5)**

**Cognitive level/CGAS**					

Mental retardation	170^Δ^	155 (91)	15 (9)	0.2 (0.1-0.4)***	0.4 (0.3-0.8)**

CGAS		40.9 (SD 12.2)	41.9 (SD 6.2)	1.0 (0.99-1.02)	-″

Prevalence of several vulnerability factors, and Relative Risk (Hazard ratio) estimated by univariate and multivariate Cox Regression for the study population of convicted (n = 131) and not convicted (n = 410) during the follow-up period. Significant Relative Risk are given in bold (* p < 0. 05, ** p <0. 01, *** p <0. 001). Variables obtaining a p-value < 0.05 in the unadjusted analyses were entered into the adjusted analysis.

-″ = The variable was not entered into the multivariate analyses (p > 0.05).

ns = non significant

▫ = The variable was not entered into the multivariate analysis because of overlapping construct with the MR variable.

^Δ ^= Includes those with mental retardation **only**.

**Figure 1:**
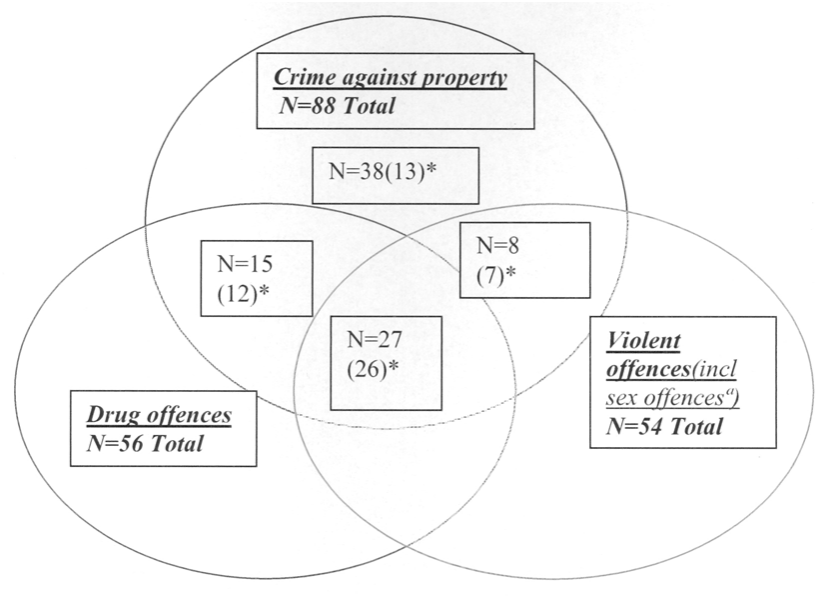
**Distribution and overlap of the various crimes committed in the convicted group, n = 131**. *ª *: 9 sexual offences, 4 of these sexual offences only. ( )* = in combination with "other crimes". (Ex: 10 (2)*= 2 of ten offences in combination with "other crimes".) Those committed only "other crimes" (n = 14), are excluded from the diagram.

Although the crimes were of different types, they all showed extensive overlap (Figure 1). Most offenders had committed crime against property (n = 88, 67%), followed by drug offences (n = 56, 43%) and violent offences (n = 54, 41%). The mean number of sentences was 4.7 (SD 5.5, range 1-35), and the median was 2.0.

Fifteen (11%) participants, who had only received judicial fines, had committed a variety of crimes. Those receiving unconditional or conditional sentences were significantly younger at their first offence than those who only received judicial fines (20.1 years; SD 5.9, range 14-46 years, vs. 25.7 years, SD 6.8, range 16-37 years, p < 0.01).

None of the offenders was sentenced to mandatory care.

### Childhood precursors of convictions for delinquency

Table 2 shows the relationship between possible vulnerability factors recorded at baseline and convictions recorded at follow-up. Results from univariate and multivariate analyses performed are presented.

Three child psychiatric disorders were positively associated with a criminal act in the univariate Cox regression analyses (Table 2). Conduct disorder (56% convicted, RR = 3.2, 95% CI = 2.0-4.9), hyperkinetic conduct disorder (54% convicted, RR = 3.4, 95% CI = 2.2-5.2) and mixed disorder of conduct and emotions (33% convicted, RR = 1.6, 95% CI = 1.1-2.5) represented significantly higher risk of later criminality when compared with the other mental disorders. The diagnoses of PDD (6% convicted, RR = 0.2, 95% CI = 0.1-0.4) and MRO (3% convicted, RR = 0.1, 95% CI = 0.0-0.9) significantly reduced the risk of future criminal behaviour. Male gender (31% convicted, RR = 3.8, 95% CI = 2.3-6.3) and CFD scale score (RR = 1.4, 95% CI = 1.2-1.6) were other vulnerability factors representing higher risk of later criminality, while MR (9% convicted, RR = 0.2, 95%CI = 0.1-0.4) represented lower risk of later criminality. All but one of the eight associated factors were entered into a Cox regression. Because the MRO variable represented overlapping constructs with the overall MR variable, it was not included in the equation. Six variables remained significant in the final multivariate model (Table 2): Conduct disorder (RR = 2.0, 95%CI = 1.2-3.4), hyperkinetic conduct disorder (RR = 2.7, 95% CI = 1.6-4.4), male gender (RR = 3.6, 95%CI = 2.1-6.1) and CFD scale score (RR = 1.3, 95% CI = 1.1-1.5) represented higher risk for later delinquency. The diagnosis of PDD (RR = 0.4, 95%CI = 0.2-0.9) and MR (RR = 0.4, 95%CI = 0.3-0.8) reduced the risk for later conviction.

We found no evidence of interactions. We specifically found no interaction between cognitive level and ADHD or between CD and chronic family difficulties (data not shown).

We also forced the ADHD variable into the final model. The results of main vulnerability factors did not change (data not shown).

Because a significant proportion of the participants had mental retardation (n = 170), and because the cognitive level was different between diagnostic groups (Table 1), separate Cox analyses were used to evaluate those with normal cognitive abilities. We found the relative strengths of the main vulnerability factors for criminality to be similar (data not shown). We also ran analyses where sub-grouping of mental retardation was more fine-meshed, and where those with severe mental retardation (n = 73) were excluded, without changing the results of main vulnerability factors (data not shown).

Because few of the females had been convicted (n = 17), and because the gender distribution was different between diagnostic groups (Table 1), separate Cox analyses were also performed exclusively for males. In the multivariate Cox regression analyses, all the main vulnerability factors remained significant (data not shown).

Finally, when the five children in the ADHD group that only fulfilled the criteria for ADD were excluded from the ADHD group, the results remained the same. There was still no association between ADHD and delinquency.

## Discussion

In the present study, 131 (24%) individuals had committed crimes during the follow-up period and were found in the crime registry. We found conduct disorder and hyperkinetic conduct disorder in childhood to be highly associated with delinquency in adulthood. When conduct disorder was combined with emotional disorder, the association was no longer significant, and there was no direct association from ADHD in childhood to future delinquent behaviour. Thus, our two hypotheses were not confirmed. A high chronic family difficulties scale (CFD) score enhanced prediction of future criminality.

### Crime rate

The crime rate in our child psychiatric in-patient population was 24%. It is difficult to obtain reliable figures concerning the prevalence of convicted persons in Norway, but estimates indicate close to 10% [28]. Recently, in a Norwegian birth cohort from 1977, about 10% (16% males and 3% females) were charged for a crime before the age of 25 years [29]. Our findings, thus, indicate a substantial increased criminal activity in the study population compared to the general population. The increased crime rate is similar to findings in a Swedish register study of child psychiatric in-patients, 18 years or younger, of whom 21% had received sentences for criminal offences at follow-up when they were from 33 to 37 years old [30]. The delinquency rate was even higher (52%) in a long-term follow-up study of former adolescent psychiatric in-patients conducted by Kjelsberg et al. In this study, 1276 patients aged from 12 to 18 years were followed up 15-33 years after hospitalization [28]. Engqvist and Rydelius [31] found, likewise, in their study of former child and adolescent psychiatric patients, that 44% of the 279 in-patients were contained in the crime register at follow-up. The lower crime rate in our group might be due to its heterogeneous diagnostic distribution, there being a significant proportion of participants with PDD (21%). Many of our participants had cognitive level below 70 (n = 170, 31%), and when they were excluded, the crime rate in our population increased to 32%. However, regardless of these study populations being different, the main conclusion is the same: Former child and adolescent psychiatric patients are at increased risk for development of future delinquency compared to the general population.

### Childhood precursors of convictions for delinquency

#### Mental health (ICD-10)

Conduct disorder and hyperkinetic conduct disorder independently represented high risks of later court convictions. Our findings reinforce an already extensive body of research that has documented the association between early conduct problems and later delinquency [3,11,13,31-33], and give support to the assumption that conduct disorder, as an antecedent, should be a priority prevention target. For those children who met criteria for both conduct disorder and ADHD, this did not tend to add substantial to the prediction of future criminality. This is in line with Lahey et al. [34], who found no elevated risk for later antisocial problems among children who met criteria for both conduct disorder and ADHD compared to children with conduct disorder alone. However, this issue is controversial, with research showing discrepant findings [34,35], and should be further explored in future research on larger populations than the present one.

Individuals with ADHD in the absence of conduct disorder had no increased risk of delinquency compared to others in this study. Several previous studies have concluded likewise, that hyperactivity-impulsivity and attentional problems are precursors of later delinquency only when there are concurrent conduct problems [13,17,34]. Recently, Diamantopolou et al. [2], similarly, found support for this assertion, about no direct association between ADHD symptoms in early childhood and conduct problems in adolescence, in their study testing developmental pathways to antisocial personality problems.

Other studies have concluded differently, finding childhood ADHD to predict antisocial behaviour also in the absence of childhood conduct disorder [3,7,8,15].

The above mentioned findings are in many ways difficult to reconcile because the relevant studies discussed have used different designs (epidemiological vs. clinical), different classification systems (DSM-IV vs. ICD-10 vs. dimensionally scored symptoms) and different outcome measures (conviction rate, self-reported crime, antisocial personality disorder). In some of the studies [15,16] oppositional defiant disorder (ODD) was not an exclusion criteria for children with ADHD, which in turn may have increased the risk for later criminality in these ADHD groups.

Our findings partly support both of the contradictory assertions mentioned above. We did not find elevated risk for convictions among individuals with ADHD when compared to other child psychiatric patients, but the crime rates for children with ADHD seemed to be elevated compared to a crude estimate in the general population. Because the comparison group in this study was referred in-patients with other diagnoses than ADHD, our findings apply only to differences among in-patient children who received different diagnoses. Longitudinal studies including large groups of children with ADHD matched with symptom-free control groups have to be conducted in order to address the question of whether ADHD alone predicts criminality. Nonetheless, previous research has found a linear association between the number of behavioural problems and later antisocial problems [34,36]. All our children were severely affected in-patients with extensive symptom load and with low psychosocial functioning. Thus, our finding, that ADHD did not predict subsequent delinquency, should strengthen the assumption that there is no direct association between ADHD and later criminality.

However, early hyperkinetic symptoms have been reported to enhance the development of early onset CD [35]. Our findings thus illuminate the importance of target intervention in the ADHD group, to prevent development of comorbid conduct disorder, which has been claimed to be the mediator between ADHD and criminality [32].

Despite children in our in-patient population displaying highly elevated levels of symptoms, co-occurring internalizing and externalizing problems did not appear to elevate risk for developing delinquency. We found that mixed disorder of conduct and emotions was less likely to be associated with delinquency than pure conduct disorder. Recently, Diamantopoulou et al. [2], similarly, found that neither depression nor somatic problems in adolescence appeared to add to the prediction of adult antisocial problems. This contrasts with the results of a study conducted by Sourander [8], who found that children with combined emotional and conduct problems had a higher risk of criminality than those with conduct problems only. Fombonne and Harrington found, on their side, similar outcome in children with comorbid depressive and conduct disorder and in children with conduct disorder alone [18,19]. Our discrepant findings may be due to different study designs. Sourander and Diamantopoulou conducted epidemiological studies in which internalizing and externalizing problems at baseline were measured using self-reports and reports from parents and teachers, without any clinical diagnostic evaluation of the samples. In the present clinical study, symptom patterns were classified according to standardized diagnostic criteria. Although Fombonne and Harrington also used a categorical approach in their clinical studies, they focused on depression. In our study, we clustered all emotional disorders into a single group, and we cannot, therefore, directly compare these studies. We need further large scale intervention studies to finally answer whether targeting emotional disorders is likely to reduce the association between conduct disorder and delinquency.

#### Sociodemographic variables

Well known risk factors such as male gender and family adversities [3,28,37] were also in this study associated with later delinquency. We used the chronic family difficulties scale (CFD) score to assess the family adversities. A high CFD total score, representing an accumulation of unfavourable psychosocial background factors (e.g. low family income, poor social network and parental psychopathology), significantly predicted future criminality (p < 0.01).

Previous studies have reported low income families with disturbed environments to be prevalent among children with conduct disorders [3,38]. Recently, D'Onofrio 2009 et al. [39] even claimed that there is a causal association between family income and childhood conduct problems, and emphasized the importance of identifying family income as a crucial risk factor for development of early CD. In our study, high CFD scores were highly prevalent among all the children with conduct disorders (Table 1), and about half of these children turned out to be delinquent. The present finding highlights the importance of early intervention among children with severe family difficulties to avoid development of early CD, which is highly associated with further criminality.

#### Factors reducing the risk of delinquency

As demonstrated in other studies, PDD and mental retardation appeared to protect against delinquency [26,40]. This is not unexpected considering the overlap between the PDD group and those with mental retardation (66% of the PDD population had mental retardation), and that mentally retarded and autistic people are often raised in protected environments at home or in institutions. Besides, individuals with severe mental retardation are seldom prosecuted for violation of the law, although, according to the penal code, they can be sentenced to mandatory care. Thus, the strong negative association between cognitive disabilities and convictions found in this study may therefore be an artefact of such practices. Worth mentioning, when those individuals with severe mental retardation were excluded from the material, mental retardation still remained protective, which means that having a mild mental retardation also seemed to reduce the risk for criminality in our population. The strong negative association between PDD and convictions was found regardless of exclusion of those with comorbid mental retardation. This strengthens the finding of reduced risk for delinquency in this group regardless of intellectual level, but may still be due to close monitoring of these individuals in protected environments.

### Strengths and limitations

In this study, data were collected over a period varying from 19 to 41 years (the mean follow-up period was 30 years) in a longitudinal follow-up study to examine the link between psychiatric disorders in childhood and later delinquency. The study's strengths are the long follow-up interval and the large number of patients included, combined with the high proportion of patients traced at follow-up (87%). The outcome measures are robust official records data. To a certain extent, the study's design can be regarded as quasi-prospective because the data were collected from the hospital records before the outcome were obtained from official records.

The study has several limitations. All information was based on hospital records; these are not always reliable scientific sources. However, the hospital records were of good quality giving a detailed and thorough description of the patients' symptoms, scholastic skills and childhood circumstances. The re-diagnosis and scoring of the data from the study sample were completed by experienced psychiatrists. Inter-rater reliability was high, in line with previous research, where validity of file-based diagnostic ratings has been found satisfactory [41,42].

The study population is not representative of child psychiatric patients in general. Because these in-patients represented severe cases that might represent a worsening of the long-term outcome, factors identified in this study should only be interpreted as vulnerability factors within a psychiatric in-patient population. However, we have confidence in the findings because they replicate results from other studies with different populations. Results obtained from multivariate regression analyses should always be interpreted with caution, and the factors identified should not be interpreted as causative factors.

The study considered only sentenced criminality. This probably underestimates the antisocial activity, perhaps especially among those individuals with severe mental retardation which often are exempted from criminal prosecution. However, the use of official crime records ensured that only criminal acts severe enough to elicit sanctions from the justice system were included, and bias in self-reported offending were avoided.

Generalization of the findings is limited to nations with similar criminal judicial systems.

Finally, our small group of convicted females provided insufficient statistical power to predict delinquent behaviour in females exclusively.

## Conclusions

Our results indicate that it seems possible to identify children with a high risk of developing delinquency. The crime rate in this study of former child psychiatric in-patients was more than twofold that of the general population. We found conduct disorder alone or in combination with hyperactivity in childhood to be highly related to delinquency in adulthood. Our controversial finding, that conduct disorder combined with emotional disorder was less associated than conduct disorder alone, should be addressed in future research. We found chronic family difficulties to predict future criminality. Taking both diagnosis and family difficulties into account could enhance the prediction of future delinquency.

Interestingly, children with ADHD in the absence of conduct disorder had no higher risk for later delinquency than the rest of the study population in the present study. As these children had extensive symptom load, our finding strengthens the assumption that there is no direct association between hyperkinetic symptoms and criminality.

## Competing interests

The authors declare that they have no competing interests.

## Authors' contributions

All authors (except BS) conceived of and designed the study. MM participated in the collection of data, performed statistical analyses and drafted the first manuscript. BG participated in the collection of data, helped with statistical analyses and made significant contribution to the final draft. BS made significant contribution to the statistical analyses and critically reviewed the manuscript. EK made significant contribution to the final draft. AMM participated in the collection of data, made significant contribution to the final draft and supervised the work and critically reviewed the manuscript. All authors read and approved the final manuscript.

## Pre-publication history

The pre-publication history for this paper can be accessed here:

http://www.biomedcentral.com/1471-244X/11/57/prepub
